# From East to West – an early-career researcher’s voyage across continents

**DOI:** 10.7554/eLife.113141

**Published:** 2026-09-29

**Authors:** Samuel C Eziuzor

**Affiliations:** 1 School of Engineering, University of British Columbia - Okanagan Kelowna Canada

**Keywords:** research culture, funding, postdoc, transferable skills, employment, early-career researcher, None

## Abstract

One scholarship can open a research career. It can also start a years-long negotiation with loneliness, ambiguous job titles, and the quiet arithmetic of underfunded postdoctoral life. An early-career researcher who has made the journey from West Africa to East Germany and on to Western Canada describes what rarely makes it into the acceptance letter, and what institutions on both sides of the Atlantic could do differently.

The email that changes a research career rarely looks dramatic but can be a turning point for the recipient. For most, it arrives as a routine notification from the German Academic Exchange Service, the DAAD, confirming a fully funded doctoral scholarship. But for early-career researchers (ECRs) – in particular from West Africa – that kind of letter is often the start of a much longer story, one that runs from underfunded labs at home and the disciplined intensity of a doctorate in a foreign country, to the uncertain contract economy of a postdoc and debates about whether and where to settle.

## Why leave at all?

Limited research infrastructure and scarce funding push many West African scientists to seek doctoral training abroad. Germany has become one of the more visible destinations: its public universities charge no tuition, and scholarship schemes such as the DAAD cover stipends, health insurance, and often a flight home. Between 2017 and 2022, more than 7,000 students from West Africa were enrolled in doctoral programmes in Germany, according to the UNESCO Institute for Statistics (UNESCO Institute for Statistics, 2023). For many, the scholarship letter is the easy part.

Most doctoral programmes in Germany are taught in English, but life outside the university is not. While seminars, supervision meetings, and conference talks happen in a language ECRs already speak fluently, tenancy contracts, tax forms, and the weekly grocery run do not. By the author’s own account, “Navigating daily tasks like grocery shopping…” was an unexpectedly steep part of the learning curve, one with no academic credit attached, but a real measure of how quickly one may start to feel at home. The gap between the language of the lab and the language of the street can quietly erode the sense of belonging that long doctoral training depends on.

## The cost of independence

The German doctorate is structurally generous, typically three to four years of funded, largely self-directed research, but unforgiving of the wrong expectations. Supervisors expect doctoral researchers to set their own course early in their academic training – a culture that rewards initiative but can feel like abandonment to scholars trained in more hierarchical systems. “The expectation to work independently was both empowering and overwhelming…” is how the author describes the adjustment. The same independence that accelerates a thesis can also isolate the writer.

This isolation has a measurable cost. A 2017 survey by Levecque and colleagues found that roughly one in two PhD students experiences psychological distress, and one in three is at risk of a diagnosable psychiatric disorder (Levecque et al., 2017). The COVID-19 pandemic compounded this: a study conducted during Germany’s lockdown found doctoral researchers reporting lower satisfaction with their training and overall wellbeing during that period (Naumann et al., 2022); a reminder of how thin the margins already were before the pandemic layered isolation on top of isolation.

## A second uprooting built on uncertain ground

For West African ECRs who complete a German PhD and remain in research, the next step is often to move west again, to pursue a postdoctoral fellowship. For example, Canada, with its multicultural research hubs and funding schemes such as Mitacs, Canada’s national non-profit research and innovation organisation, and the Banting Postdoctoral Fellowships (Mitacs funds research partnerships between universities and industry; the Banting is Canada’s most competitive postdoctoral award)**,** offers a different kind of collaborative opportunity. For many, Canada represents a chance to lead independent projects and build a research identity distinct from that of a doctoral supervisor. For West African ECRs trained in German universities, Canada offers a convergence of advantages that few alternatives match: research conducted in English, immigration pathways that treat a postdoctoral appointment as a viable route towards permanent residency, and funding structures – particularly Mitacs – designed to build industry-academia partnerships and, with them, exposure to careers outside the tenure track.

However, such an opportunity can sit on uncertain institutional footing. Postdoctoral researchers in Canada frequently hold ambiguous employment classifications – neither student nor regular employee – which translates into inconsistent access to benefits (Mitacs, 2023a). According to Mitacs, in 2023, roughly two-thirds of Canadian postdocs earned less than CAD 45,000 a year, and many lacked health or dental coverage (Mitacs, 2023a). Career development support is similarly thin: more than half receive no training in project management, intellectual property, or other skills relevant to careers outside academia (Mitacs, 2023b). “The lack of clear career pathways and inadequate support made the postdoctoral phase particularly stressful…” the author reports, a frustration less about the science itself than the structure surrounding it.

## Carrying the weight twice

**Figure 1.**
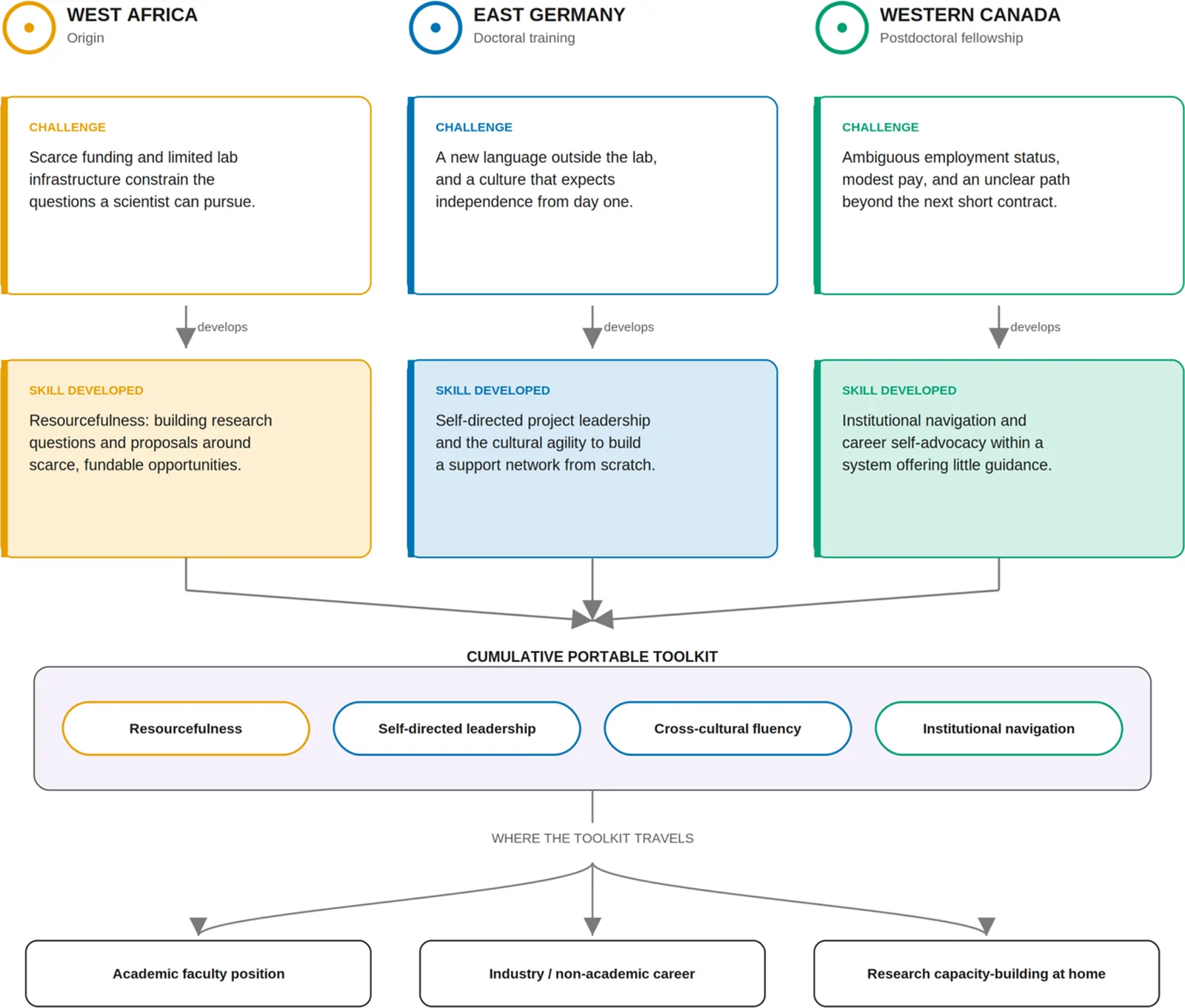
Hardship as curriculum: the portable toolkit built across three career stages. Each relocation imposes a distinct hardship but also forges a transferable skill that compounds into a portable professional toolkit. The diagram traces three career stages, leaving West Africa, doctoral training in East Germany, and a postdoctoral fellowship in Western Canada, showing the principal challenge encountered at each stage (top row, outlined) and the skill or strategy it produced (bottom row, shaded). These four competencies combine into a cumulative toolkit applicable to an academic faculty position, a non-academic career, or a return to research capacity-building in the researcher’s home country.

The mental-health toll documented in Germany does not disappear on arrival in Canada; if anything, precarity compounds it. Often, it is the cumulative effect of two consecutive precarious career stages, each demanding excellence while offering little structural security in return (Figure 1). A national survey by the Canadian Association of Postdoctoral Scholars found that 79% of postdocs report mental health challenges, including feelings of being overwhelmed, loneliness, and anxiety (Canadian Science Policy Centre, 2024). Nearly a third report experiencing harassment, discrimination, or bullying, with many incidents going unreported for fear of career repercussions (Canadian Science Policy Centre, 2024).

## What would make the difference?

Most ECRs who make this journey still want to do research, in a permanent academic post, in industry, or back home, building research capacity in the countries they left. The scarcity of tenure-track positions and limited exposure to non-academic career paths make academic and industry careers harder than they should be. Returning to West Africa to run local research groups faces additional barriers: the same under-resourced infrastructure and funding gaps that prompted departure in the first place have rarely improved in the interim.

Three changes would help almost immediately: according to Mitacs, standardising postdoctoral employment classifications so that access to benefits does not depend on which institution administers the contract (Mitacs, 2023a); building transferable-skills training into postdoctoral programmes as a matter of course rather than an optional extra (Mitacs, 2023b); and treating mental health support as core research infrastructure rather than an afterthought, accessible without first requiring an ECR to name and justify what they are struggling with.

None of this diminishes what the journey makes possible: access to better-resourced laboratories, mentorship, and a global research network that would be difficult to build from home alone. But the scholarships and fellowships that make the journey possible rarely come with a manual on what happens after the funding letter arrives: the language barriers, the isolation, the ambiguous job titles, a second precarity layered on the first. Closing that gap is not a matter of charity toward international researchers. Rather, it is a question of willingness of the recruiting institutions to support the careers they are asking researchers to build.

## References

[bib1] (2024). All is not well with Canada’s postdoctoral scholars: current challenges and solutions for researcher well-being. https://sciencepolicy.ca/posts/all-is-not-well-with-canadas-postdoctoral-scholars-current-challenges-and-solutions-for-researcher-well-being/.

[bib2] Levecque K, Anseel F, De Beuckelaer A, Van der Heyden J, Gisle L (2017). Work organization and mental health problems in PhD students. Research Policy.

[bib3] (2023a). University Affairs: Low pay and ambiguous job status frustrates Canadian postdocs. https://www.mitacs.ca/news/university-affairs-low-pay-and-ambiguous-job-status-frustrates-canadian-postdocs/.

[bib4] (2023b). Postdocs in Canada face mismatch between skills training and future careers. https://www.mitacs.ca/news/postdocs-in-canada-face-mismatch-between-skills-training-and-future-careers/.

[bib5] Naumann S, Matyjek M, Bögl K, Dziobek I (2022). Doctoral researchers’ mental health and PhD training satisfaction during the German COVID-19 lockdown: results from an international research sample. Scientific Reports.

[bib6] (2023). Global Flow of Tertiary-Level Students: Inbound and Outbound International Mobility by ISCED Level. United Nations Educational, Scientific and Cultural Organization.

